# Maternal Haploids Are Preferentially Induced by *CENH3*-*tailswap* Transgenic Complementation in Maize

**DOI:** 10.3389/fpls.2016.00414

**Published:** 2016-03-31

**Authors:** Timothy Kelliher, Dakota Starr, Wenling Wang, Jamie McCuiston, Heng Zhong, Michael L. Nuccio, Barry Martin

**Affiliations:** Biology Research, Syngenta Crop Protection, Research Triangle ParkDurham, NC, USA

**Keywords:** CENH3, centromeres, haploid induction, doubled haploids, genome elimination, mutant complementation, RNA interference

## Abstract

Doubled haploid plants are invaluable breeding tools but many crop species are recalcitrant to available haploid induction techniques. To test if haploid inducer lines can be engineered into crops, *CENH3*^−∕−^ and *CENH3:RNAi* lines were complemented by *AcGREEN-tailswap-CENH3* or *AcGREEN-CENH3* transgenes. Haploid induction rates were determined following testcrosses to wild-type plants after independently controlling for inducer parent sex and transgene zygosity. CENH3 fusion proteins were localized to centromeres and did not cause vegetative defects or male sterility. *CENH3*:*RNAi* lines did not demonstrate consistent knockdown and rarely produced haploids. In contrast, many of the complemented *CENH3*^−∕−^ lines produced haploids at low frequencies. The rate of gynogenic haploid induction reached a maximum of 3.6% in several hemizygous individuals when backcrossed as males. These results demonstrate that *CENH3*-*tailswap* transgenes can be used to engineer *in vivo* haploid induction systems into maize plants.

## Introduction

Doubled haploid (DH) generations facilitate production of pure homozygous seed in one generation, enabling breeders to bypass the seven or eight seasons of self-pollination that are normally required to produce inbred germplasm (Wêdzony et al., 2009). DH plants offer a shortcut to complete genome homozygosity, and they are valuable tools for trait introgression, QTL mapping, and direct cytoplasmic conversions (Chang and Coe, 2009). The DH process is initiated with the induction of haploid (1N) plants. Haploid plants are typically sterile because meiotic success requires homologous chromosome pairing. For haploids to set seed, floral cell lineages must undergo doubling (DNA replication without mitosis), which can be induced via treatment with a microtubule polymerization inhibitor such as colchicine. Doubled haploid cell lineages have two identical copies of a single genome, and self-pollinating DH plants set 100% inbred seed. While many crops in the cereals, brassicas and cucurbits are bred through DH generations, several important crop species remain recalcitrant to standard haploid induction techniques (Kasha and Maluszynski, 2003).

Haploid plants are typically induced *in vitro* via gametophytic embryogenesis or *in vivo* through haploid induction (HI) crosses.In the former, anthers, microspores or ovaries are cultured into pro-embryos that spontaneously develop into doubled haploid plants (Barnabás et al., 1999; Seguı'-Simarro and Nuez, 2007). In the latter, a line of interest is crossed to a haploid inducer line, and after fertilization, a fraction of the embryos develop as haploids containing only the genome of interest (Qiu et al., 2014). While the mechanism varies in different systems, the process often involves elimination of the inducer genome in early embryogenesis (Zhang et al., 2008).

The developmental genetics underlying genome elimination is not well-understood. The process was recently engineered via heterologous complementation of *CENTROMERIC HISTONE3* (*CENH3*) with tail-altered versions of the same gene (Ravi and Chan, 2010). *CENH3* is the centromere-specific variant of *HISTONE3* (*H3*) and is required for kinetochore nucleation and spindle attachment in mitosis and meiosis. *CENH3* is composed of two domains. The DNA-binding histone fold domain (HFD) is conserved across higher eukaryotes and is very similar to the same domain in *H3*. In contrast, the N-terminal tail domain varies widely among closely related species and has been hypothesized to be involved in speciation (Maheshwari et al., 2015). Ravi and Chan produced *Arabidopsis cenh3/cenh3* lines complemented by *CENH3-tailswap* transgenes in which the N-terminal tail was swapped with the shorter *H3* tail. These lines produced little pollen and set predominantly diploid seed after self-pollination.

However, when these lines were backcrossed by wild-type pollen, the female *tailswap* chromosomes were frequently lost post-fertilization, resulting in the production of ~30% androgenic haploids (in which the male genome was maintained while the female genome was lost), as well as many aneuploids in which the female genome was partially lost. The authors reasoned that centromeres constructed with tail-altered CENH3 proteins function normally until they are forced to compete with wild-type centromeres for centromere loading with kinetochore components in the hybrid zygote and early embryo. This causes reduced spindle attachment of the inducer genome, leading to elimination of those chromosomes via fragmentation, and micronuclei formation during mitosis. It has remained unclear why haploids were found more frequently when the altered CENH3 was on the female side of the backcross (~30%) than the male (4%), but incomplete penetrance is a common feature of genome elimination in other induction systems (Zhao et al., 2013).

Worldwide the maize hybrid seed market is supported by industrial DH pipelines that utilize intra-specific *in vivo* haploid inducer lines derived from Stock6 (Coe, 1959). In contrast to *CENH3*-based induction, Stock6 and its derivative lines only act as haploid inducers when they are used as the male parent in the induction cross (i.e., induced gynogenesis) (Xu et al., 2013). As with *CENH3*-based induction, it is thought that the male inducer chromosomes are eliminated post-fertilization in early embryo development. Maize DH production is characterized by high rates of embryo abortion and low seed set, likely due to instability of the inducer chromosomes during the development of the embryo and endosperm (Zhang et al., 2008). These inefficiencies increase DH production costs and limit the rate at which new inbred lines can be developed.

In the present study *ZmCENH3* knock-down and knock-out lines were complemented with tail-altered *ZmCENH3* transgenes, and haploid induction rates (HIR) were measured following reciprocal backcrosses to wild-type. Twelve *CENH3* complementation strategies were tested by independently controlling for parent sex and transgene zygosity in the context of two tail sequences. While many of the strategies led to modest levels of HI, one had a strong effect, which may provide guidance for future endeavors in *CENH3* engineering.

## Methods

### Crossing strategy

Two approaches were undertaken to eliminate native *CENH3* - a native gene knockout method, and an RNAi method. In the latter, a dsRNA hairpin targeting the native *CENH3* hyper-variable tail sequence was added to the *AcGREEN-CENH3* and *AcGREEN-tailswap-CENH3* transgenic constructs. To avoid RNAi targeting, the *CENH3* transgene tail sequences were codon-altered. T0 transformants made in the NP2222 background were self-pollinated, and homozygous T1 progeny were forwarded to haploid induction tests. A total of 21 individuals from 10 events were tested for haploid induction via reciprocal backcrosses to NP2222. Some individuals were backcrossed multiple times onto several NP2222 ears. In total, 50 ears were generated and over 5000 embryos were evaluated via transgene PCR assays to assess ploidy status.

In the native gene knockout method, the *AcGREEN-CENH3* and *AcGREEN-tailswap-CENH3* transgenes were introduced into a background heterozygous for a UniformMu allele obtained from the maize Co-op (*UFMu-01386*, http://www.maizegdb.org/uniformmu). In this allele the transposon insertion is in the first exon of the *ZmCENH3* gene (GRMZM2G158526). Though other grass species have two versions of *CENH3*, there appears to be only one in maize (Zhong et al., 2002), an assertion supported by the fact that this *cenh3* transposon insertion allele is homozygous lethal. To generate homozygous lines complemented by one of the *CENH3* transgenes, the *cenh3* allele was introgressed from the W22 donor line into NP2222 via three backcrosses. Then, *CENH3/cenh3* heterozygous BC3 plants were crossed to transgenic T0 plants carrying one of the two tail-altered versions of *CENH3*. The F1 progeny were self-pollinated and homozygous *cenh3*/*cenh3* F2 plants were selected for reproductive evaluations. All homozygous mutant plants were complemented by at least one copy of a transgene, and hemizygous and homozygous individuals were independently evaluated. A total of 10 events were tested for haploid induction via reciprocal crosses to NP2222. Several individuals were backcrossed as a male to several NP2222 ears. In total, 203 backcrossed ears were generated and nearly 17,000 embryos were extracted and tested for ploidy status. As control, several *CENH3* wild-type (+/+) plants with two copies of the *AcGREEN-tailswap-CENH3* transgene were tested for haploid induction rates. A total of 3072 control progeny were tested.

### Vector construction

Two *CENH3* fusion transgenes were synthesized: *AcGREEN-CENH3* and *AcGREEN-tailswap-CENH3*. For the *AcGREEN-tailswap-CENH3* transgene, the *CENH3* hyper-variable tail sequence was replaced with the maize *H3* tail sequence (N-MARTKHQAVRKTAEKPKKKLQF ERSGGASTSATPERAAGTGGRAASGGDSVKKTKPRHRW-). For the *AcGREEN-CENH3* transgene, the *CENH3* hyper-variable tail sequence was codon-altered to avoid RNAi knockdown, but the amino acid sequence was not changed. The partial *CENH3* sequence targeted by RNAi was (5′-ctcggc gacgccggaaagggctgctgggaccgggggaagagcggcgtctggaggtgactcagt taagaagacgaaaccacgccaccgctggcg gcca-3′). Twenty-three out of thirty-two codons in the RNAi-relevant sequence of *AcGREEN-tailswap-CENH3* were altered at the degenerate third position. Both cassettes were driven by the native *ZmCENH3* promoter, and the Clontech (http://www.clontech.com) fluorophore AcGFP1 (which we call *AcGREEN*) was fused to the N-terminus of both *CENH3* versions to establish transgenic protein localization to centromeres. For each of the two transgenes, one construct was made such that the T-DNA insert would consist of only the native promoter and the *CENH3* fusion transgene, and a second binary construct was made containing the transgene plus the RNAi hairpin designed to knock down native *CENH3*. The RNAi hairpin was driven by an ubiquitin promoter. The selectable marker was *PMI* (*phosphomannose isomerase)* and it was driven by the maize ubiquitin promoter for selection on mannose (Negrotto et al., 2000). The four constructs were transformed into Syngenta's standard transformable inbred line NP2222 using *Agrobacterium tumefaciens* callus co-culture, and at least eight single copy events each were sent to the greenhouse, grown to maturity, and self-pollinated. At least five events for each construct were selected at random for further evaluation.

### Confocal microscopy

Homozygous transgene positive T1 plants were sampled to determine if the *CENH3* transgenes were expressing and localizing properly. In at least two individuals from each of two events for each of the four constructs, samples were taken from the scutellum, lateral roots, and meiotic-staged anthers, fixed in 100% ethanol, and stained with propidium iodide (PI) using established techniques (Kelliher and Walbot, 2011). Anthers were then sectioned or imaged directly on a Zeiss laser scanning microscope (LSM 710). The *AcGREEN* fluorophore was visualized at 488 nm and the emission spectra was collected at 520–550 nm, while PI was excited at 560 nm and the emission spectra was collected at 600–650 nm. Images were captured using an AxioCam MRc camera and processed in ZEN 2011 image analysis software.

### qRT-PCR

Meiotic staged anthers were sampled from T1 homozygous *CENH3:RNAi* plants to measure transcript knockdown by qRT-PCR. Prior work had established that anthers about 2.0 mm long contain hundreds of reproductive cells in meiosis (Kelliher and Walbot, 2011). Three lower floret anthers at this stage were dissected from central rachis and side branch spikelets about 2 days prior to tassel emergence from the whorl. Samples were taken in quadruplicate and transcript abundance was measured using a quantitative reverse transcriptase PCR (qRT-PCR) reaction with primers specific to the native *CENH3* allele, 5′-GCGACGCCGGAAAGG-3′ and 5′-TGGCGTGGTTTCGTCTTCTTA-3′. Transcript abundance was compared to an endogenous control to standardize for starting cDNA amounts.

### Ploidy evaluation

In both the knockout and RNAi strategies, selected lines were evaluated for reproductive fitness and reciprocally backcrossed to NP2222 for haploid induction tests. Fourteen to seventeen days after pollination, ears were harvested and viable and aborted kernels were counted. In most cases, 84 embryos were then extracted (although in a few cases, 168 embryos were extracted). After extraction, embryos were placed on solid media and allowed to germinate a rudimentary shoot during 3–7 days incubations in the dark (Supplementary Figure 1A). The root and lower part of the scutellum and embryo proper were then sampled (Supplementary Figure 1B) and tested for ploidy via PCR assay, while the remaining tissues (scutellum, embryo, and shoot) were stored at –80°C for ploidy confirmation via Flow Cytometry (ploidy analysis). Putative haploids were identified by PCR reactions that detected the transgenes and/or the *cenh3* allele. For the knockout strategy, diploids were identified as having one copy of the *cenh3* allele, while putative haploids were identified as having zero copies of the *cenh3* allele (because the inducer genome is lost during haploid induction). For the RNAi strategy, diploids were identified by having one copy of the transgene, while putative haploids were identified by having zero copies of the transgene. Because aneuploidy or pollen contamination could easily lead to false scoring using these methods, all putative haploids were tested by Flow Cytometry on a Partec Ploidy Analyzer with UV lamp to detect DAPI stained, extracted nuclei. Samples were sliced using a razor blade for 30 s, extracted for 1 min in nuclei extraction buffer, and incubated for 5 min in DAPI stain. The gain was set at 525, the speed was set at 4 ul/sec, and the sample concentration was established to be 2000–3000 cells/mL. The limits used to categorize ploidy status were: Haploid, 1st peak 90–110, 2nd peak 180–220; Aneuploid, 1st peak 110–190, 2nd peak 220–380; Diploid, 1st peak 190–210, 2nd peak 380–420.

### Statistical analyses

Haploid induction rates were compared to the background rate in maize by a two-tailed Z-test for comparing two proportions. The background rate was established to be 0.065% (see Table 1). For evaluation of RNAi efficacy, relative transcript abundance for each sample was compared by Student's *T*-test to the average of the four control samples on the same plate.

**Table 1:** **Haploid induction frequency determination in control (***CENH3 +/+***) lines complemented by two copies of the ***AcGREEN-tailswap-CENH3*** transgene**.

**Event**	**Outcrossed as male**					**Outcrossed as female**				
	**Crosses**	**Embryos**	**Diploids**	**Haploids**		**Crosses**	**Embryos**	**Diploids**	**Haploids**	
***CENH3*** **+/+;** ***AcGREEN-tailswap-CENH3*** **+/+**										
A004A	8	672	672	0		4	331	331	0	
A034A	6	499	498	1	Total: 0.05% HIR	3	250	250	0	Total: 0.10% HIR
B013A	6	502	502	0		2	164	164	0	
B024A	5	402	402	0		3	252	251	1	
Totals	25	2075	2074	1		12	997	996	1	

*The average induction frequency in these crosses was 0.065% (2 haploids out of 3072 embryos)*.

## Results

**Figure 1:**
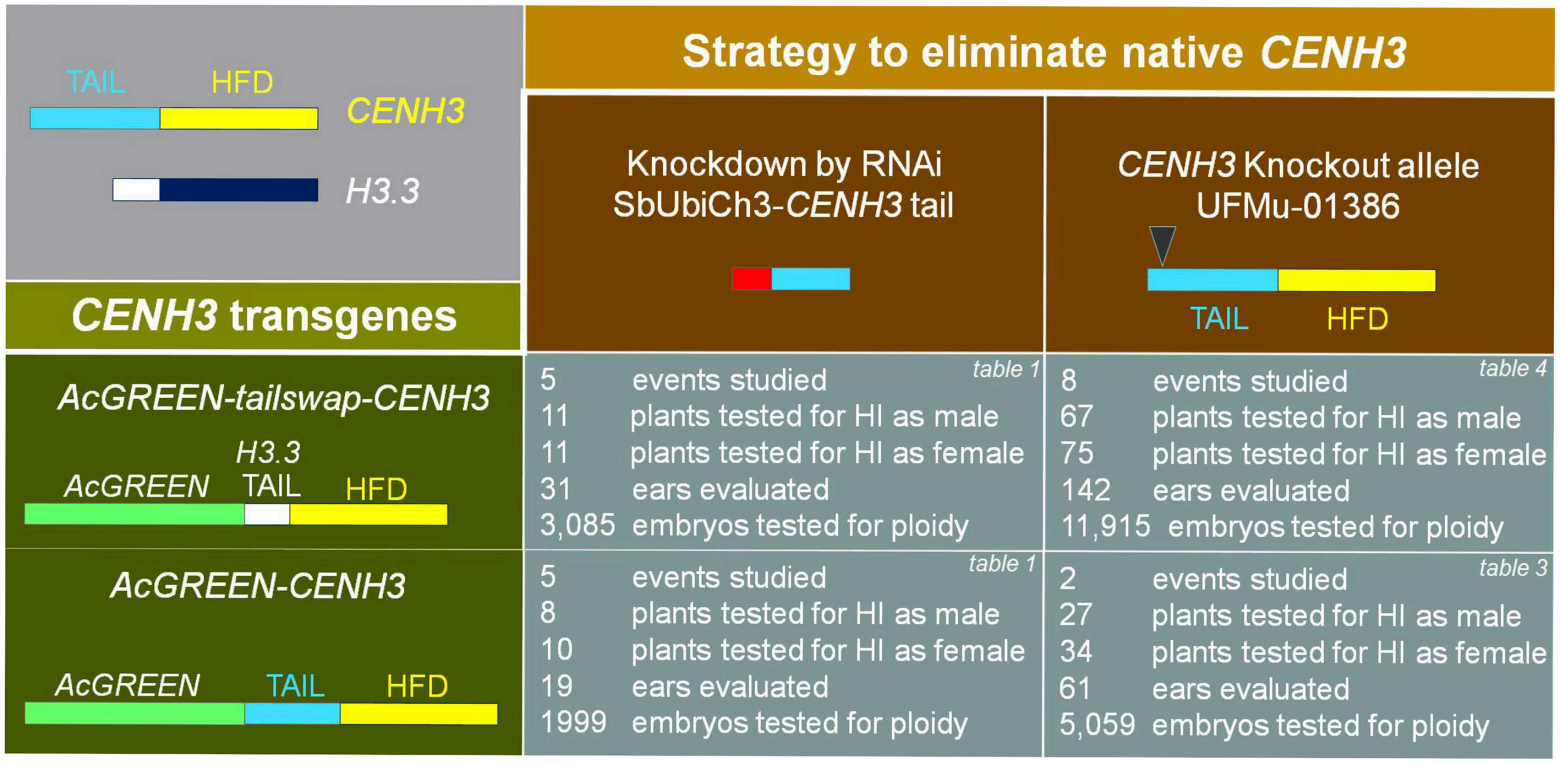
**Experimental overview of the four ***CENH3*** complementation strategies tested**. Two native *CENH3* elimination methods (**top row**) were paired with each of the two transgenes (**left column**). The number of transgenic events studied, plants crossed, and progeny screened for each strategy is shown in the four gray boxes (**bottom right**). In the top right corner of these boxes, the corresponding data table for that box's strategy is indicated. Not included in this diagram is the experimental design for the control data measuring the background haploid induction rate. The experimental set up for that test is described in the methods, and the data is found in Table 1.

Two tail-altered *CENH3* transgenes, *AcGREEN-CENH3* and *AcGREEN-tailswap-CENH3*, were each tested with two native gene complementation strategies (Figure 1). In the first strategy binary vectors were constructed containing one of the two transgenes plus a dsRNA hairpin cassette designed to target the *CENH3* tail in native transcripts only. In the second elimination strategy, transgenic events lacking the dsRNA hairpin were introgressed into a *cenh3*/*cenh3* background. Figure 1 summarizes the numbers of events, individual plants, and progeny tested for each approach. In total, 20 events, 243 crosses, and 22,000 embryos were screened.

**Figure 2:**
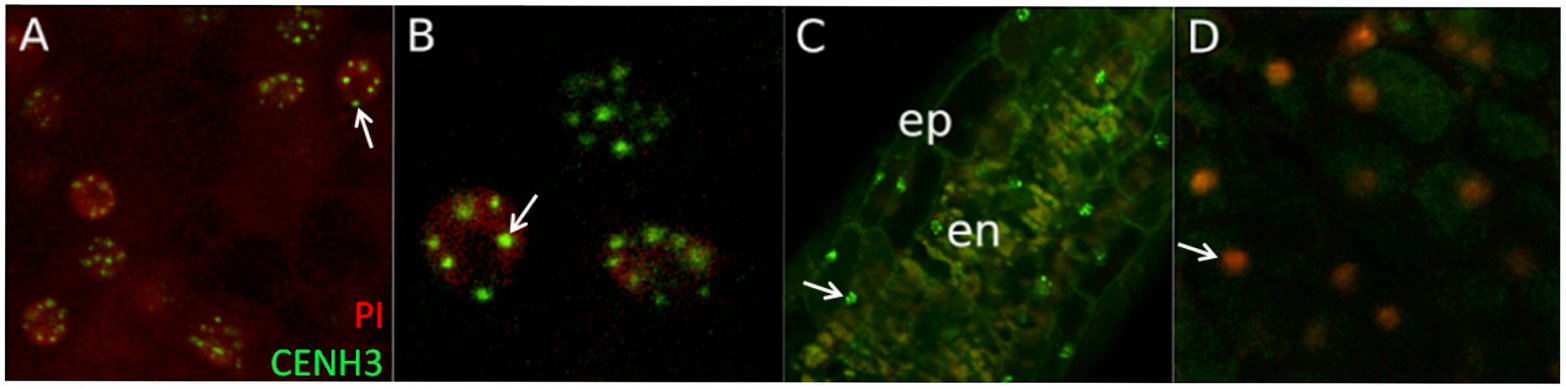
*****AcGREEN-tailswap-CENH3*** localizes to centromeres**. These images are representative of those found in all events checked from all four constructs. **(A)** CENH3 localization has a punctate pattern in the scutellar nuclei of a transgene-hemizygous individual from event ^*^32A. Arrow indicates a nucleus containing several CENH3+ foci corresponding to centromeres. **(B)** Close up of **(A)**. Arrow indicates one CENH3+ centromere. **(C)** CENH3 localizes to nuclei in the epidermal and endothecial cells of the pre-meiotic (~1.0 mm long) anther from a transgene-hemizygous individual from event ^*^32A. Arrow indicates one epidermal cell nucleus with several discrete CENH3+ centromeres. **(D)** CENH3 was not observed in a transgene-null individual from event ^*^32A. Arrow indicates one scutellar nucleus with no CENH3+ centromeres visible. *PI*, propidium iodide; *ep*, epidermal cells; *en*, endothecial cells.

To establish that the fusion proteins localized to centromeres, one transgene-negative and two transgene-positive T1 individuals were imaged from at least two events from each of the four constructs. In every transgene-positive sample checked, multiple cells in the scutellum (Figures 2A,B), and meiotic staged anther (Figure 2C) had several intra-nuclear *AcGREEN+* spots, consistent with a centromeric localization. This pattern was not found in any of the negative control samples checked (Figure 2D), but it was found in transgene-hemizygous and -homozygous individuals, regardless of the presence or absence of the native *CENH3* wild-type allele. Only ~40% of nuclei in a given tissue sample had this pattern, while the remaining nuclei did not have any fluorescent signal in the *AcGREEN* channel. This is consistent with the idea that *CENH3* loading on centromeres is cell-cycle dependent. In most nuclei that had the signal, between 8 and 10 dots were clearly distinguished, but some nuclei had up to 20 dots, most likely due to endo-reduplication or because those cells were in the G2 phase of the cell cycle.

To establish the control rate of haploid induction in our transgenic plant population, plants homozygous for wild-type *CENH3* were used in induction tests. *CENH3*/*CENH3* F2 plants homozygous for the *AcGREEN-tailswap-CENH3* transgene were identified from each of four events and 37 backcrosses were made to wild-type (25 through the male, and 12 through the female). Two haploids were found among the 3072 progeny tested (Table 1). With this level of investigation, the control induction rate was thus found to be ~0.065%, which falls in the reported background range of gynogenic haploid induction (0.05–0.1%; Chang and Coe, 2009).

**Figure 3:**
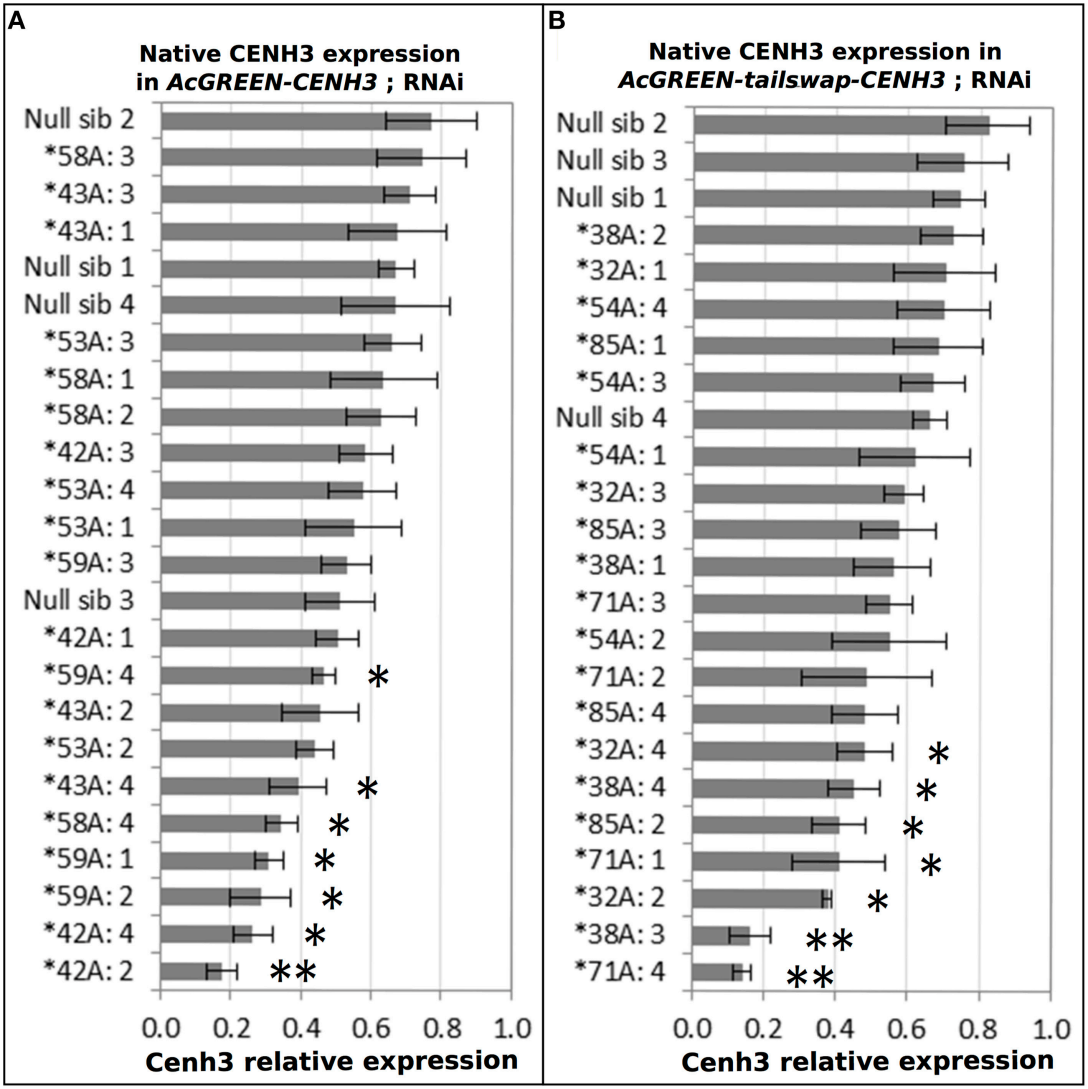
*****Cenh3*** knockdown by RNAi**. Five events from each RNAi construct were grown and four transgene-homozygous individuals from each were sampled to measure the extent of *Cenh3* knockdown. Four transgene-negative siblings from each construct were sampled and tested for comparison (Null sib 1-4). Samples were analyzed by qRT-PCR using a primer pair specific to the *CENH3* tail. The X-axis is a ratio of *Cenh3* expression to the endogenous control. Individuals were tested in triplicate and error bars indicate the standard deviation in the replicates. Results are displayed in rank order with the highest relative expression at the top of each chart. **(A)** Native CENH3 expression levels in individuals from AcGREEN-CENH3 + CENH3:RNAi events. **(B)** Native Cenh3 expression levels in individuals from AcGREEN-tailswap-CENH3 + CENH3:RNAi events. In total, fourteen individuals had significant *Cenh3* knockdown (^*^p < 0.01; ^**^p < 0.001) and these, along with a few selected others, were forwarded to reproductive evaluations and haploid induction tests.

The impact of the dsRNA hairpin transgenic cassette on native *CENH3* expression was evaluated by qRT-PCR in the T1 generation of five events for each construct. Homozygous T1 plants were compared to siblings lacking the transgenes. Meiotic staged anthers were chosen as the tissue to evaluate *Cenh3* knockdown because they contain active mitosis and meiosis, both of which require *CENH3*. To ensure a statistically sound assessment of transcript abundance, four homozygous plants from each event were tested in triplicate. A majority of the plants exhibited only a slight down-regulation of *Cenh3* mRNA compared to controls (Figures 3A,B). Fourteen individuals were significantly knocked down (*p* < 0.01) and had mRNA levels between 21 and 65% of the average of the controls. Ten *AcGREEN-tailswap-CENH3* and nine *AcGREEN-CENH3* plants with strong knockdown, and one unaffected individual from each construct, were forwarded to reproductive evaluations and haploid induction tests through reciprocal backcrosses to wild-type.

In the haploid induction tests of the RNAi lines complemented by *AcGREEN-tailswap-CENH3*, just 5 haploids were found out of 3084 embryos tested, for an average induction rate of 0.16% (Table 2), just slightly higher than the established background HIR for maize, which was 0.05–0.1% (Chang and Coe, 2009). Considering only the crosses in which the transgene was on the male side, the rate of induction was 0.24% (4/1669), which was not significantly higher than background (*Z* = 1.62, *p* > 0.05). One homozygous plant exhibited a haploid induction rate of 2.4% when outcrossed as a male (4/168 progeny were haploids). That plant (^*^ 71A:4) also had the highest knockdown by RNAi, as the *Cenh3* transcript abundance was equal to 18.4% of the average of the within-plate controls. The other haploid embryo was induced androgenically by an individual from a different event. For the RNAi line complemented by *AcGREEN-CENH3*, only one, female-induced haploid was found out of nearly 2000 embryos tested (Table 2).

**Table 2:** **Haploid induction frequency determination for ***CENH3*** RNAi lines complemented by ***AcGREEN-tailswap-CENH3*** (top) and ***AcGREEN-CENH3*** (bottom) transgenes**.

	**Outcrossed as Male**					**Outcrossed as Female**				
**Event**	**Crosses**	**Embryos**	**Diploids**	**Haploids**		**Crosses**	**Embryos**	**Diploids**	**Haploids**	
***CENH3:RNAi; AcGREEN-tailswap-CENH3*** **+/+**										
^*^ 32A	4	335	335	0		2	336	336	0	Total: 0.07% HIR
^*^ 38A	6	502	502	0	Total: 0.24% HIR	2	167	167	0	
^*^ 54A	2	165	165	0		2	167	166	1	
^*^ 71A	6	503	499	4		3	499	499	0	
^*^ 85A	2	164	164	0		2	247	247	0	
Totals	20	1669	1665	4		11	1416	1415	1	
***CENH3:RNAi; AcGREEN-CENH3*** **+/+**										
^*^ 43A	2	165	165	0		2	335	334	1	Total: 0.08% HIR
^*^ 53A	3	250	250	0	Total: 0.0% HIR	2	252	252	0	
^*^ 58A	No ears available					1	83	83	0	
^*^ 42A	2	162	162	0		3	251	251	0	
^*^ 59A	2	167	167	0		2	334	334	0	
Totals	9	744	744	0		10	1255	1254	1	

Most individuals were selected because they exhibited significant Cenh3 knockdown, yet very few haploids were found. Two individuals from event ^*^42A had less than 30% of wild-type Cenh3, but neither plant produced haploids. When the AcGREEN-tailswap-CENH3 event ^*^71A:4 was outcrossed as a male, a 2.4% HIR was found (4 haploids out of 168 embryos).

In the *CENH3* knockout strategy, *AcGREEN-tailswap-CENH3* and *AcGREEN-CENH3* T0 plants were crossed to a line heterozygous for *cenh3*, a recessive, transposon-insertion allele of *CENH3* that is homozygous embryo lethal. Transgene-positive F1 plants carrying *cenh3* were self-pollinated, and F2 progeny homozygous for *cenh3* were tested for transgene zygosity status, as determined by T-DNA specific assays compared to endogenous controls (Supplementary Table S1). Among thousands of F2 progeny tested, all *cenh3*/*cenh3* plants were complemented by at least one copy of a *CENH3* transgene, confirming that *cenh3* is homozygous lethal. Dozens of *cenh3*/*cenh3* plants hemizygous or homozygous for one of the transgenes developed normally, indicating that the transgenes were functional and complemented the knock-out allele *in planta*.

To assess the HIR in *CENH3* complementation lines, *cenh3/cenh3* F2 individuals that were complemented with one of the two transgenes were reciprocally crossed to wild-type. Progeny were tested for ploidy status using PCR tests outlined in the Methods Section. Putative haploids were confirmed by ploidy analysis on a flow cytometer. In total, 203 crosses were made from 8 *AcGREEN-tailswap-CENH3* and 2 *AcGREEN-CENH3* events. Results were sorted by transgene zygosity (i.e., hemizygous or homozygous) and sex of the transgenic parent. There were clear differences in the HIR obtained depending on each of these factors (Tables 3, 4).

**Table 3:** **Haploid induction frequency determination in ***CENH3***^**−∕−**^ lines complemented by one (top) or two (bottom) ***AcGREEN-CENH3*** copies**.

	**Outcrossed as male**					**Outcrossed as female**				
**Event**	**Crosses**	**Embryos**	**Diploids**	**Haploids**		**Crosses**	**Embryos**	**Diploids**	**Haploids**	
***CENH3***^−−∕−−^**;** ***AcGREEN-CENH3*** **hemizygous**										
A003A	8	663	661	2	Total: 0.31% HIR	17	1406	1405	1	Total: 0.15% HIR
A026A	11	915	912	3		7	582	580	2	
Totals	19	1578	1573	5		24	1988	1985	3	
***CENH3***^−∕−^**;** ***AcGREEN-CENH3*** **homozygous**										
A003A	3	252	252	0	Total: 0.15% HIR	5	418	417	1	Total: 0.12% HIR
A026A	5	408	407	1		5	415	415	0	
Totals	8	660	659	1		10	833	832	1	

*The HIR when the transgene was hemizygous on the male side of the cross was 0.31%, which is significantly higher than background (Z = 2.10, p < 0.05). All other crossing strategies produced haploids at more modest rates*.

**Table 4:** **Haploid induction frequency determination in ***CENH3***^**−∕−**^ lines complemented by one (top) or two (bottom) copies of the ***AcGREEN-tailswap-CENH3*** transgene**.

	**Outcrossed as male**					**Outcrossed as female**				
**Event**	**Crosses**	**Embryos**	***D***	***H***	**Max HIR found**	**Crosses**	**Embryos**	***D***	***H***	**Max HIR found**
***CENH3***^−∕−^**;** ***AcGREEN-tailswap-CENH3*** **hemizygous**										
A004A	5	416	409	7	3.6% (3/84)	7	583	583	0	
A005A	6	504	502	2	1.2% (1/84)	3	251	251	0	
A008A	3	250	250	0		8	659	655	4	2.4% (2/84)
A034A	7	584	580	4	3.6% (3/84)	11	915	912	3	1.2% (1/84)
B004A	3	244	240	4	3.6% (3/84)	7	582	582	0	
B013A	6	501	497	4	3.6% (3/84)	6	501	500	1	1.2% (1/84)
B019A	3	252	251	1	1.2% (1/84)	3	252	252	0	
B024A	6	497	491	6	2.4% (2/84)	3	248	248	0	
Totals	39	3248	3220	28	0.86% HIR	48	3991	3983	8	0.20% HIR
***CENH3***^−∕−^**;** ***AcGREEN-tailswap-CENH3*** **homozygous**										
A005A	7	588	588	0		6	504	504	0	
A008A	6	500	499	1	1.2% (1/84)	3	252	252	0	
A034A	2	168	168	0		2	165	164	1	1.2% (1/84)
B004A	4	330	330	0		4	323	322	1	1.2% (1/84)
B013A	3	252	251	1	1.2% (1/84)	4	333	333	0	
B024A	6	504	503	1	1.2% (1/84)	8	753	752	1	1.2% (1/84)
Totals	28	2342	2339	3	0.13% HIR	27	2330	2327	3	0.13% HIR

*The columns labeled “D” refer to the diploid embryo count. The columns labeled “H” refer to the haploid embryo count. The columns labeled “Max HIR found” refer to the highest rate of haploid induction found among all the crosses made using individuals from the given event. The average induction frequency when the transgenic line was hemizygous on the male side of the cross was 0.86%, which is significantly higher than background (Z = 4.61, p < 0.01). All other crossing strategies produced haploids at rates that were not significantly different compared to controls*.

Specifically, low induction rates were found when the *AcGREEN-CENH3* transgene was homozygous on both the female side of the cross (1 haploid out of 833 progeny) and the male side of the cross (one haploid out of 660 progeny) (Table 3). The HIR increased when hemizygous individuals were tested. These lines induced 3 haploids out of 1988 embryos when employed as female inducers, and 5 haploids out of 1578 embryos when used as pollen donors (0.31% HIR) (Table 3). This represents a statistically significant 4.9-fold increase over the control.

**Figure 4:**
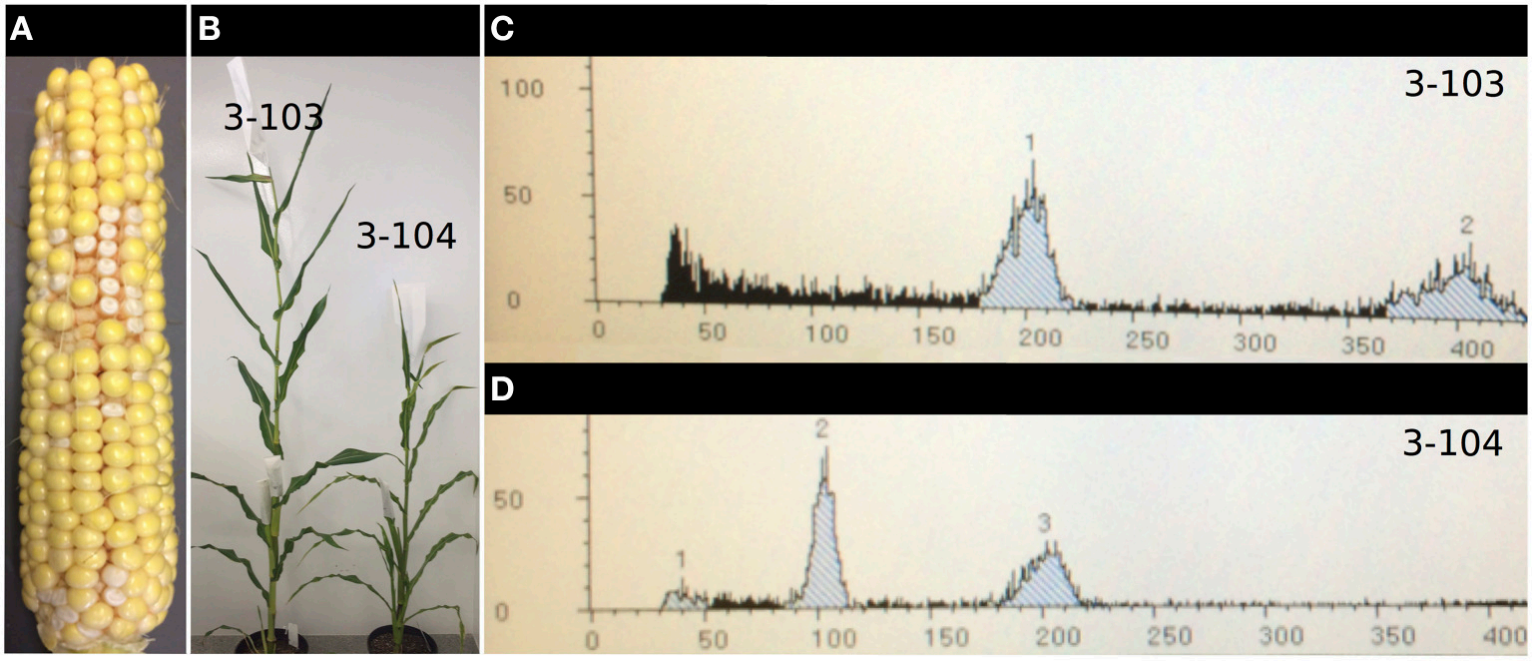
*****CENH3***-based haploid induction in maize. (A)** Photograph of the ear crossed by pollen from individual ^*^ A004A:3, which exhibited a 3.6% haploid induction rate (3 haploids found out of 84 embryos). Such elevated levels of induction were always found coincident with low levels of embryo abortion (the white kernels in the photo) scattered around the ear. **(B)** Progeny from this ear were germinated on media, sampled, and identified by PCR as either haploid or diploid. Based on the PCR calls, a few putative haploid and diploid plants were then transplanted to soil and grown until the diploid plants were shedding pollen. Putative haploids (such as plant ^*^ A004A:3-104, right) were male and female sterile. They were also shorter and had thinner leaves than the putative diploids (such as plant ^*^ A004A:3-103, left). **(C,D)** Adult leaf samples were tested to confirm ploidy status. The diploid 2N peak was set at 200 units (*X-axis*) by adjusting the gain while running cells extracted from a known diploid plant through the flow cube. **(C)** The sample from the putative diploid plant ^*^A004A:3-103 had a 2N peak at 200 units and a smaller 4N peak at 400 units. **(D)** The putative haploid plant ^*^A004A:3-104 had a 2N peak at 100 units and a smaller 4N peak at 200 units. The image in **(D)** is representative of the histograms found for all putative haploids.

Slightly elevated induction rates were found when the *AcGREEN-tailswap-CENH3* line was homozygous on either side of the induction cross (3/2330 as a female and 3/2342 as a male) (Table 4). Similarly, when this transgene was hemizygous and used as the female in the induction cross, a total of 8 out of 3991 embryos were identified as haploids, for an HIR of 0.20%, which is about three-fold above the background. When the *AcGREEN-tailswap-CENH3* transgene was used as the male in the induction cross, 28 confirmed haploids were recovered out of 3252 embryos, for an average HIR of 0.86% (Table 4). This represents a statistically significant 13.2-fold increase over the control. The highest HIR on an ear was 3.6%, which was found four times in this set. One of the ears, a cross by pollen from individual ^*^A004A:3, was photographed and showed ~11% embryo abortion (43/390 kernels) (Figure 4A). Some progeny from this ear were germinated and an example of a haploid and diploid were photographed at maturity (Figure 4B). Their ploidy status was confirmed via Flow Cytometry (Figures 4C,D).

In the *Arabidopsis* study, a high level of aneuploidy was found among the progeny, likely a result of partial chromosome elimination during haploid induction. In contrast, all 60 of the maize haploids we identified by PCR assay were confirmed as true haploids via ploidy analysis. We also screened 96 putative diploids in the ploidy analyzer, specifically sampling them from ears that had haploid induction occurring. Of these 96 putative diploids, 95 were scored as true diploids via ploidy analysis, while one embryo was scored as an aneuploid. The aneuploid was from event ^*^ A008A, when the transgene was in a hemizygous state on the female side of the cross. Several other putative diploids sampled from the same ear were confirmed as diploids.

## Discussion

Doubled haploid generations allow breeders to bypass years of inbred line generation by single seed decent. For crops that do not have reliable haploid production methods, the 2010 Ravi and Chan study suggested that engineered haploid induction could be available for all crops. In an attempt to demonstrate the validity of that theory, we tested several biotechnological approaches toward engineering *CENH3-*based haploid induction in maize. Although, the rates of haploid induction were not overwhelming compared to industry-standard maize haploid inducer lines, which have been bred for high rates of induction (8–15%), we were able to demonstrate several instances of induction rates as high as 3.6% in some crosses. Such rates were found most frequently when the *AcGREEN-tailswap-CENH3* transgene was hemizygous on the male side of the cross in the *cenh3*/*cenh3* background. However, from individual to individual, the haploid induction rate varied widely and on average, the male, hemizygous induction crosses induced haploids at a frequency of 0.86%, which is 13.2-fold higher than background.

While this rate was much higher than the other reproductive strategies tested in this study (Table 5), it is still relatively low compared to the native Stock6 induction system (Coe, 1959). It is unclear why the hemizygous trasngenics consistently performed better than their homozygous counterparts. One reasonable conjecture is that it has to do with the abundance of the transgenic CENH3 protein in the absence of wild-type *CENH3*. One might imagine that the less transgenic CENH3 available, the higher the frequency of faulty centromere construction. Until now the only plant in which *CENH3* alteration led to haploid induction was *Arabidopsis*. Delayed loading of *CENH3* onto centromeres was shown to be concurrent with post-zygotic genome elimination in barley *vulgare* × *bulbosum* wide crosses (Sanei et al., 2011), but the present work represents the first example of engineered *CENH3-*based haploid induction in a monocotyledonous crop plant.

**Table 5:** **Summary of haploid induction rate data following outcrosses with ***CENH3***-altered transgenic maize lines**.

**Inducer transgene**	**Native CENH3**	**Inducer sex**	**Transgene Zygosity**	***C***	***E***	***H***	**Avg. HIR**	**Best HIR**	**FC over BG**
*AcGREEN-tailswap-CENH3*	−∕−	Male	Hemizygous	39	3252	28	0.86%	3.6%	13.2 X^**^
*AcGREEN-CENH3*	−∕−	Male	Hemizygous	19	1578	5	0.32%	~1.2%	4.9 X^*^
*CENH3:RNAi + AcGREEN-tailswap-CENH3*	+∕+	Male	Homozygous	20	1669	4	0.24%	2.4%	3.7 X
*AcGREEN-tailswap-CENH3*	−∕−	Female	Hemizygous	48	3991	8	0.20%	3.6%	3.1 X
*AcGREEN-CENH3*	−∕−	Female	Hemizygous	24	1988	3	0.15%	~1.2%	2.3 X
*AcGREEN-CENH3*	−∕−	Male	Homozygous	8	660	1	0.15%	~1.2%	2.3 X
*AcGREEN-tailswap-CENH3*	−∕−	Male	Homozygous	28	2342	3	0.13%	~1.2%	2.0 X
*AcGREEN-tailswap-CENH3*	−∕−	Female	Homozygous	27	2330	3	0.13%	~1.2%	2.0 X
*AcGREEN-CENH3*	−∕−	Female	Homozygous	10	833	1	0.12%	~1.2%	—
*CENH3:RNAi + AcGREEN-CENH3*	+∕+	Female	Homozygous	10	1255	1	0.08%	~1.2%	—
*CENH3:RNAi + AcGREEN-tailswap-CENH3*	+∕+	Female	Homozygous	11	1416	1	0.07%	~1.2%	—
*CENH3:RNAi + AcGREEN-CENH3*	+∕+	Male	Homozygous	9	744	0	0.00%	—	—
*AcGREEN-tailswap-CENH3*	+/+	Combined	Homozygous	37	3072	2	0.065%	~1.2%	Control

Induction strategies are sorted by the type of transgene used (left column), the native CENH3 locus zygosity status (second column), the inducer parent sex in the directional cross (third column), and transgene zygosity (fourth column). The column labeled “C” is the number of crosses made, “E” is the number of embryos extracted, and “H” is the number of haploids recovered. The categories are presented in rank order with the highest average HIR at the top of the table. The “best HIR” column refers to the highest HIR seen in a single cross in each category. “FC over BC” refers to the fold change of the induction rate over the background (control) rate as determined in Table 1. The top two induction strategies exhibited a significantly higher induction rate compared to control using a Z-test for comparing two means (

* p < 0.01;

** *p < 0.001)*.

*Both of these strategies were based on hemizygous complementation of the CENH3 knockout allele and outcrosses through the male*.

There were significant differences between the outcomes of the *Arabidopsis* work and the present study. While more events and crosses were tested here, the highest HIR was significantly lower than in *Arabidopsis* (Ravi and Chan, 2010). In addition, the most frequent type of haploid induction seen in maize was gynogenetic induction, in which male inducer chromosomes are lost and female chromosomes are retained. Only rarely was the converse seen—when the female line was the inducer and the male chromosomes were retained. High male sterility limited study of *CENH3*-based gynogenesis in *Arabidopsis*, where a 3% HIR was found using male inducers, compared to 30% with female inducers (Ravi and Chan, 2010). It is not clear what accounts for the difference between the species, or if a lack of androgenetic haploidy will be a common feature for other monocotyledonous crops. There is a natural androgenetic mutation in maize called *indeterminate gametophyte1* (*ig1*) (Evans, 2007). It would be interesting to determine if induction rates increase when *CENH3-tailswap* technology is tested in an *ig1* background.

There are several biotechnological strategies for genetic complementation in plants. In this study we tested two methods to complement native *CENH3* with the tail-altered transgenes. In the RNAi approach binary constructs were made containing both the tail-altered transgenes and a dsRNA designed to specifically knock down native *Cenh3* transcript. This method offered the advantage of evaluating haploid induction rates in the T1 generation. We used meiosis-staged qRT-PCR to select homozygous T1 plants with the greatest extent of knockdown for forwarding to haploid induction tests. Unfortunately neither dsRNA construct induced consistent knock down, and overall, the RNAi strategy failed to induce haploids at a significantly higher rate than the background level. The one plant that induced haploids at a modest rate (2.4%) was also the plant with the strongest knockdown, but others with similar levels of knockdown did not induce haploids in the embryos tested. It is reasonable to infer that haploid induction rates will be low unless the native CENH3 protein product is either significantly reduced or eliminated. A multi-copy RNAi line may perform better than the single-copy line used in this study.

While the transgenic plants in this study did not induce haploids as frequently as in *Arabidopsis* (Ravi and Chan, 2010), neither did they produce nearly as many aneuploid embryos, nor did they experience a dramatic loss of male fertility. We consistently observed a low level of embryo abortion on ears (ranging from 1 to 12%) including many ears that had haploid progeny. As with the native maize Stock6 induction system, it is possible that kernel abortion is a product of partial or complete inducer chromosome elimination in the endosperm, which would produce a ploidy imbalance, a known source of endosperm failure. Based on these observations, it would be interesting to test in the future whether embryo rescue leads to higher haploid recovery rates.

While partial female sterility was observed on some ears, abundant pollen was produced in all events and there were no instances of partial male sterility. In *Arabidopsis*, high male and female sterility accompanied haploid induction, and pollen was pooled from several individuals just to make a single outcross. The low pollen load of the transgenics was later associated with the GFP tag, which caused CENH3 depletion in meiotic centromeres resulting in chromosome misalignment and the production of aneuploid microspores (Ravi et al., 2011). It is unclear why similar levels of sterility did not occur in this study, but it fits with a trend seen in other systems: Lower rates of haploid induction are associated with fewer pleiotropic reproductive defects.

One way in which the data presented here was consistent with the *Arabidopsis* work was that no haploids were found that retained chromosomes from the transgenic parent. Consistently, it appears that when a “wild-type” genome and a genome derived from a tail-altered *CENH3* complementation line are hybridized, it is the genome derived from the altered *CENH3* line that is lost, regardless of the sex of the parent. This observation, along with evolutionary studies of the *CENH3* tail sequence, led Maheshwari et al. (2015) to hypothesize that *CENH3* is involved in the development of centromere specific reproductive barriers during speciation. The authors tested this hypothesis with an elegant transgenic experiment, in which native *AtCENH3* was complemented by orthologs from *Lepidium oleraceum, Brassica napa*, and *Zea mays*. These *CENH3* complementation plants developed normally and were self-fertile, but outcrosses resulted in low rates (1–11%) of haploids, aneuploids, and other genomic rearrangements. It appears that in both monocots and dicots, genes with divergent *CENH3* tail sequences are complementary in developmental contexts outside of hybridization.

Recently, the state of *CENH3* technology was advanced by work showing that single nucleotide polymorphisms (SNPs) in the histone fold domain are sufficient to induce haploids in *Arabidopsis* (Karimi-Ashtiyani et al., 2015; Kuppu et al., 2015). Heterologous complementation was then used to show that such SNPs can cause centromere disruption in barley and sugar beet (Karimi-Ashtiyani et al., 2015), but haploid induction tests of barley TILLING alleles did not succeed, likely because knock-out mutations were only available for the β copy of *CENH3* (Karimi-Ashtiyani et al., 2015), while the putatively redundant α copy was still functional.

In the present study, the events with the highest HIR produced multiple haploids in some crosses but none in others. For instance, in events ^*^ A034A and ^*^ B004A, several hemizygous F2 plants were crossed as males, and while one cross from each event produced three haploids, other crosses produced zero. Because the haploid induction tests in this study were performed on an F2 population generated by hybridization of NP2222 with a W22/NP2222 BC3 line, such variation may reflect segregation of genetic elements that modify the induction rate. In addition, the genomic context of the T-DNA insertion could contribute to event-by-event HIR variation.

## Author contributions

TK: Experimental design, confocal analysis, crossing, qRT-PCR, embryo extraction, data analysis, manuscript writing. DS: Plant care, sampling for qRT-PCR, crossing, embryo extraction, manuscript editing. WW: Embryo PCR and analysis for determining haploid induction rates, manuscript editing. JM: Embryo PCR and analysis for determining haploid induction rates, manuscript editing. HZ: Producing and analyzing transformation events, manuscript editing. MN: Experimental design, construct design, manuscript editing. BM: Experimental design, project sponsorship, manuscript editing.

## Funding

Internal (Syngenta) funding.

### Conflict of interest statement

The authors declare that the research was conducted in the absence of any commercial or financial relationships that could be construed as a potential conflict of interest. The reviewer (Dr. Kumlehn) and handling editor declared their shared affiliation, and the handling editor states that the process nevertheless met the standards of a fair and objective review.

## Abbreviations

HIR: haploid induction rate (the number of haploid progeny as a proportion of total progeny in a haploid induction cross)
DH: doubled haploid
HI: haploid induction
*CENH3*: *CENTROMERIC HISTONE3*
*H3*: *HISTONE3*
qRT-PCR: quantitative reverse transcriptase – polymerase chain reaction
PCR: polymerase chain reaction.
