# Supplementary material for: Maternal Haploids Are Preferentially Induced by CENH3-tailswap Transgenic Complementation in Maize
Source: Front Plant Sci. 2016 Mar 31;7:414. doi: 10.3389/fpls.2016.00414 (PMC4814585; doi:10.3389/fpls.2016.00414)
Supplement: Supplementary file 1 [file Table1.DOCX]

**Table 1. Example of PCR assay data table used to identify *cenh3* homozygous, transgene positive F2 individuals for haploid induction tests.** This table is from one of the *CENH3* knockout, transgenically-complemented F2 families we evaluated to identify and select individuals that were homozygous for the *cenh3* allele assay (far right column; homozygous call = 2) and either hemizygous (call=1) or homozygous (call=2) for the three transgene assays in the left columns, including the *PMI* selectable marker, the UBI promoter, and the *AcGREEN-CENH3* transgene. Rows highlighted in yellow are transgene hemizygous and *cenh3* homozygous; rows highlighted in blue are transgene homozygous and *cenh3* homozygous.

|  | PMI | | UBI Promoter | | AcGREEN-CENH3 | | *mu-cenh3* | |
| --- | --- | --- | --- | --- | --- | --- | --- | --- |
|  | Raw | Call | Raw | Call | Raw | Call | Raw | Call |
| 1-copy control | 0.84 | 1 | 1.1 | 1 | ND | ND | ND | ND |
| 1-copy control | 1.1 | 1 | 1.29 | 1 | ND | ND | ND | ND |
| 1-copy control | 1.36 | 1 | 1.13 | 1 | ND | ND | ND | ND |
| 1-copy control | 1.05 | 1 | 1.31 | 1 | ND | ND | ND | ND |
| 2-copy control | ND | ND | 1.89 | Hom | ND | ND | ND | ND |
| 2-copy control | ND | ND | 2.15 | Hom | ND | ND | ND | ND |
| 2-copy control | ND | ND | 1.79 | Hom | ND | ND | ND | ND |
| 2-copy control | ND | ND | 2.17 | Hom | ND | ND | ND | ND |
| WT | 0 | 0 | 0 | 0 | 0 | 0 | 0 | 0 |
| WT | 0 | 0 | 0 | 0 | 0 | 0 | 0 | 0 |
| WT | 0 | 0 | 0 | 0 | 0 | 0 | 0 | 0 |
| WT | 0 | 0 | 0 | 0 | 0 | 0 | 0 | 0 |
| 1 | 0.96 | 1 | 0.94 | 1 | 1.13 | 1 | 0 | 0 |
| 2 | 1.84 | 2 | 1.98 | 2 | 1.59 | 2 | 0 | 0 |
| 3 | 2.02 | 2 | 2.14 | 2 | 1.98 | 2 | 0 | 0 |
| 4 | 0.92 | 1 | 1.03 | 1 | 0.89 | 1 | 0 | 0 |
| 5 | 1.12 | 1 | 0.96 | 1 | 1.51 | 2 | 0 | 0 |
| 6 | 0.94 | 1 | 1.03 | 1 | 1.01 | 1 | 1.26 | 1 |
| 7 | 1.03 | 1 | 0.93 | 1 | 1.15 | 1 | 0 | 0 |
| 8 | 1.14 | 1 | 1.14 | 1 | 0.87 | 1 | 0.92 | 1 |
| 9 | 1.09 | 1 | 0.94 | 1 | 1.03 | 1 | 0 | 0 |
| 10 | 0.89 | 1 | 0.94 | 1 | 0.95 | 1 | 0.68 | 1 |
| 11 | 0 | 0 | 0 | 0 | 0 | 0 | 0 | 0 |
| 12 | 1.01 | 1 | 0.97 | 1 | 0.95 | 1 | 2.24 | 2 |
| 13 | 1.13 | 1 | 1.26 | 1 | 0.88 | 1 | 0 | 0 |
| 14 | 1.1 | 1 | 1.01 | 1 | 1.17 | 1 | 1.75 | 2 |
| 15 | 0 | 0 | 0 | 0 | 0 | 0 | 0.73 | 1 |
| 16 | 1.11 | 1 | 1.08 | 1 | 1.06 | 1 | 0.85 | 1 |
| 17 | 1.95 | 2 | 1.97 | 2 | 1.72 | 2 | 0.69 | 1 |
| 18 | 0.91 | 1 | 1.04 | 1 | 0.79 | 1 | 0.69 | 1 |
| 19 | 1.61 | 2 | 1.79 | 2 | 1.21 | 1 | 1.15 | 1 |
| 20 | 1.92 | 2 | 1.76 | 2 | 1.58 | 2 | 1.2 | 1 |
| 21 | 1.16 | 1 | 1.13 | 1 | 1.05 | 1 | 0 | 0 |
| 22 | 0 | 0 | 0 | 0 | 0.01 | 0 | 0 | 0 |
| 23 | 0.96 | 1 | 1.06 | 1 | 1.2 | 1 | 0 | 0 |
| 24 | 2.09 | 2 | 1.78 | 2 | 2.53 | 2 | 0.8 | 1 |
| 25 | 0.95 | 1 | 1.17 | 1 | 0.79 | 1 | 0 | 0 |
| 26 | 1.81 | 2 | 1.69 | 2 | 1.53 | 2 | 0 | 0 |
| 27 | 0 | 0 | 0 | 0 | 0.01 | 0 | 0.8 | 1 |
| 28 | 0.98 | 1 | 1 | 1 | 0.83 | 1 | 0 | 0 |
| 29 | 2.19 | 2 | 2.38 | 2 | 2.09 | 2 | 2.23 | 2 |
| 30 | 1.11 | 1 | 1.17 | 1 | 1.22 | 1 | 1.88 | 2 |
| 31 | 2.01 | 2 | 1.77 | 2 | 2.45 | 2 | 0.91 | 1 |
| 32 | 1.31 | 1 | 1.18 | 1 | 1.16 | 1 | 0.82 | 1 |
| 33 | 0 | 0 | 0 | 0 | 0.02 | 0 | 0 | 0 |
| 34 | 0 | 0 | 0 | 0 | 0.01 | 0 | 0 | 0 |
| 35 | 1.88 | 2 | 1.68 | 2 | 1.43 | 2 | 0.5 | 1 |
| 36 | 1.65 | 2 | 1.9 | 2 | 2.02 | 2 | 1.07 | 1 |
| 37 | 1.08 | 1 | 1.15 | 1 | 1.47 | 2 | 1.51 | 2 |
| 38 | 1.12 | 1 | 1.1 | 1 | 0.99 | 1 | 2 | 2 |
| 39 | 1.04 | 1 | 1.02 | 1 | 0.99 | 1 | 0.9 | 1 |
| 40 | 0.97 | 1 | 0.94 | 1 | 1.24 | 1 | 0.84 | 1 |
| 41 | 1.77 | 2 | 1.59 | 2 | 1.77 | 2 | 0 | 0 |
| 42 | 1.61 | 2 | 1.74 | 2 | 1.85 | 2 | 0.63 | 1 |
| 43 | 0.84 | 1 | 1.1 | 1 | 0.93 | 1 | 0.64 | 1 |
| 44 | 1.99 | 2 | 1.89 | 2 | 1.52 | 2 | 0 | 0 |
| 45 | 0.97 | 1 | 1.15 | 1 | 1.19 | 1 | 2.33 | 2 |
| 46 | 0.99 | 1 | 1.08 | 1 | 1.03 | 1 | 0 | 0 |
| 47 | 2.22 | 2 | 1.93 | 2 | 1.87 | 2 | 0.92 | 1 |
| 48 | 1.11 | 1 | 1.06 | 1 | 1.83 | 2 | 0.96 | 1 |
| 49 | 1.62 | 2 | 2.05 | 2 | 1.48 | 2 | 0.86 | 1 |
| 50 | 1.85 | 2 | 1.85 | 2 | 1.92 | 2 | 0 | 0 |
| 51 | 0 | 0 | 0 | 0 | 0 | 0 | 0 | 0 |
| 52 | 1.63 | 2 | 1.97 | 2 | 1.67 | 2 | 0.82 | 1 |
| 53 | 1.04 | 1 | 1.03 | 1 | 1.08 | 1 | 2.01 | 2 |
| 54 | 2.1 | 2 | 2.07 | 2 | 2.02 | 2 | 1.1 | 1 |
| 55 | 0 | 0 | 0 | 0 | 0 | 0 | 1.11 | 1 |
| 56 | 1.69 | 2 | 1.64 | 2 | 1.69 | 2 | 1.74 | 2 |
| 57 | 0 | 0 | 0 | 0 | 0 | 0 | 0 | 0 |
| 58 | 0.99 | 1 | 1 | 1 | 0.87 | 1 | 0.79 | 1 |
| 59 | 1.22 | 1 | 0.93 | 1 | 1.01 | 1 | 1.07 | 1 |
| 60 | 1.08 | 1 | 1.14 | 1 | 0.88 | 1 | 0.7 | 1 |
| 61 | 1.08 | 1 | 1.04 | 1 | 1.08 | 1 | 3.01 | 2 |
| 62 | 2.13 | 2 | 2.03 | 2 | 1.93 | 2 | 0 | 0 |
| 63 | 0.99 | 1 | 1 | 1 | 1.02 | 1 | 0.93 | 1 |
| 64 | 0.83 | 1 | 1.05 | 1 | 0.96 | 1 | 0.86 | 1 |
| 65 | 1.71 | 2 | 1.73 | 2 | 1.59 | 2 | 0.9 | 1 |
| 66 | 1.93 | 2 | 1.99 | 2 | 1.95 | 2 | 1.63 | 2 |
| 67 | 1.92 | 2 | 2.05 | 2 | 1.43 | 2 | 0 | 0 |
| 68 | 0.88 | 1 | 0.92 | 1 | 0.87 | 1 | 1.05 | 1 |
| 69 | 0.79 | 1 | 0.98 | 1 | 0.84 | 1 | 1.37 | 2 |
| 70 | 0.91 | 1 | 0.89 | 1 | 0.86 | 1 | 0 | 0 |
| 71 | 0.84 | 1 | 1.04 | 1 | 0.77 | 1 | 1.03 | 1 |
| 72 | 0.89 | 1 | 1.09 | 1 | 0.79 | 1 | 1.35 | 2 |
| 73 | 1.52 | 2 | 1.6 | 2 | 1.77 | 2 | 0.75 | 1 |
| 74 | 0.75 | 1 | 0.85 | 1 | 1.01 | 1 | 0.59 | 1 |
| 75 | 0.88 | 1 | 0.79 | 1 | 0.71 | 1 | 0.62 | 1 |
| 76 | 0.87 | 1 | 0.97 | 1 | 0.88 | 1 | 1 | 1 |
| 77 | 1.01 | 1 | 0.86 | 1 | 0.93 | 1 | 1.11 | 1 |
| 78 | 0.85 | 1 | 0.83 | 1 | 0.88 | 1 | 2.58 | 2 |
| 79 | 0.79 | 1 | 0.76 | 1 | 0.86 | 1 | 1.21 | 1 |
| 80 | 1.32 | 1 or 2 | 1.55 | 2 | 1.52 | 2 | 0.97 | 1 |
| 81 | 1.44 | 2 | 1.57 | 2 | 1.62 | 2 | 0.74 | 1 |
| 82 | 1.45 | 2 | 1.7 | 2 | 1.27 | 1 | 0.86 | 1 |
| 83 | 1 | 1 | 0.85 | 1 | 1.25 | 1 | 0 | 0 |
| 84 | 0 | 0 | 0 | 0 | 0 | 0 | 1.07 | 1 |
