# Supplementary material for: Maternal Haploids Are Preferentially Induced by CENH3-tailswap Transgenic Complementation in Maize
Source: Front Plant Sci. 2016 Mar 31;7:414. doi: 10.3389/fpls.2016.00414 (PMC4814585; doi:10.3389/fpls.2016.00414)

Figure 1. Embryo germination and sampling process. A) A plate of 84 maize embryos after extraction and germination on growth media for 6 days in the dark at 22 degrees Celcius, just as was done in the manuscript as part of the ploidy analysis pipeline. B) Eight germinated shoots separated for PCR assays (right) and Flow Cytometry (left).

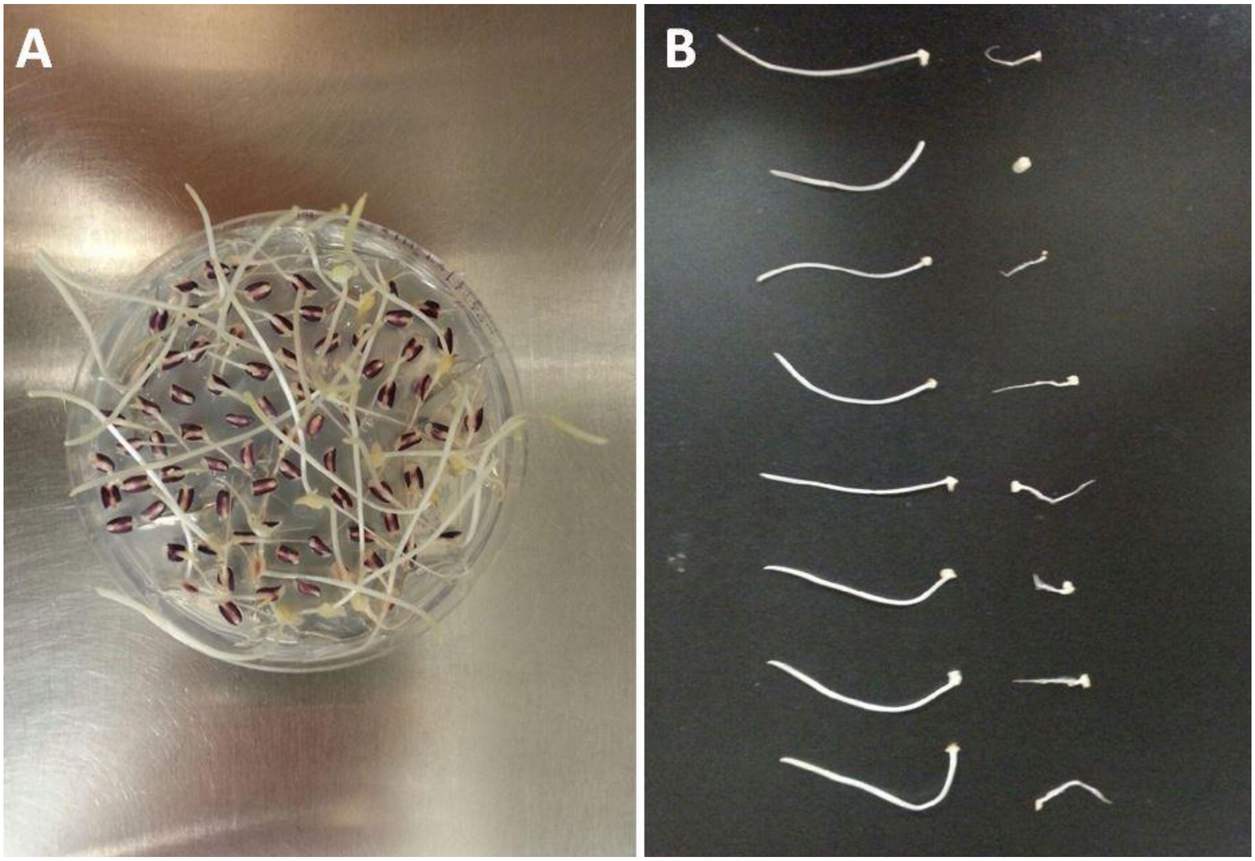

Supplement: Supplementary file 2 [file Image1.PDF]
